# Aortic Stenosis: Haemodynamic Benchmark and Metric Reliability Study

**DOI:** 10.1007/s12265-022-10350-w

**Published:** 2023-02-06

**Authors:** Harminder Gill, Joao Filipe Fernandes, Amanda Nio, Cameron Dockerill, Nili Shah, Naajia Ahmed, Jason Raymond, Shu Wang, Julio Sotelo, Jesus Urbina, Sergio Uribe, Ronak Rajani, Kawal Rhode, Pablo Lamata

**Affiliations:** 1 School of Biomedical Engineering and Imaging Sciences, King’s College London, Becket House, 1 Lambeth Palace Road, SE1 7EU London, UK; 2 Cardiology Department, Guy’s and St, Thomas’s Hospital, London, UK; 3 University of Oxford, Oxford, UK; 4 School of Biomedical Engineering, Universidad de Valparaíso, Valparaíso, Chile; 5 Biomedical Imaging Center, Pontificia Universidad Católica de Chile, Santiago, Chile; 6 Millennium Institute for Intelligent Healthcare Engineering, iHEALTH, Santiago, Chile; 7 Department of Radiology, Schools of Medicine, Pontificia Universidad Católica de Chile, Santiago, Chile; 8 Institute for Biological and Medical Engineering, Schools of Engineering, Medicine and Biological Sciences, Pontificia Universidad Católica de Chile, Santiago, Chile

**Keywords:** Haemodynamics, Aortic valve, Aortic stenosis, 3D printing

## Abstract

Aortic stenosis is a condition which is fatal if left untreated. Novel quantitative imaging techniques which better characterise transvalvular pressure drops are being developed but require refinement and validation. A customisable and cost-effective workbench valve phantom circuit capable of replicating valve mechanics and pathology was created. The reproducibility and relationship of differing haemodynamic metrics were assessed from ground truth pressure data alongside imaging compatibility. The phantom met the requirements to capture ground truth pressure data alongside ultrasound and magnetic resonance image compatibility. The reproducibility was successfully tested. The robustness of three different pressure drop metrics was assessed: whilst the peak and net pressure drops provide a robust assessment of the stenotic burden in our phantom, the peak-to-peak pressure drop is a metric that is confounded by non-valvular factors such as wave reflection. The peak-to-peak pressure drop is a metric that should be reconsidered in clinical practice.

**Graphical abstract:**

The left panel shows manufacture of low cost, functional valves. The central section demonstrates circuit layout, representative MRI and US images alongside gross valve morphologies. The right panel shows the different pressure drop metrics that were assessed for reproducibility 
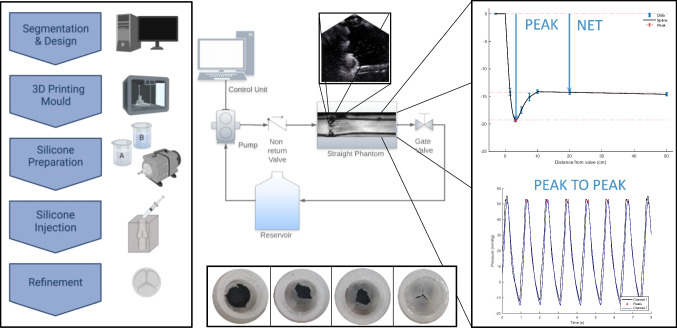

**Supplementary Information:**

The online version contains supplementary material available at 10.1007/s12265-022-10350-w.

## Introduction

### Background

Calcified and thickened aortic valve leaflets which obstruct blood exiting the left ventricle (LV) are the pathological hallmark of aortic stenosis (AS) [1]. The condition is typified by gradual restriction of the valve leaflets resulting in a long latent period from initial diagnosis to clinically relevant disease and an incidence which increases with age [2]. When left untreated, the ensuing cardiovascular morbidity and mortality are substantial [3]. Objective measures of AS severity from Doppler echocardiography (DE) such as the peak aortic velocity (Vmax), mean transaortic pressure drop (MPD, a more accurate term to the commonly used gradient) and the effective orifice area (EOA) aid in the decision to replace the valve [1, 4]. However, methods of analysing one-dimensional velocity data from DE are subject to uncontrolled sources of error, with discordant grading in 30% of cases and variable correlation with symptoms [5–7]. Gold-standard, invasive pressure measurement for haemodynamic quantification of AS severity is no longer undertaken as a first-line investigation due to incommensurate procedural risk [8]. Thus, improved, non-invasive techniques which accurately characterise the additional haemodynamic burden of AS are sought but require refinement and validation prior to clinical usage [7, 9].

### Rationale for Phantom Development

In vivo validation represents the ideal strategy to accelerate novel techniques into clinical practice. However, recruiting patients can be a difficult, costly and time-consuming process [10], especially when the severe AS cohort is old, frail and comorbid [11]. Most importantly, acquiring the desired in vivo data, the gold-standard invasive pressure measurements, carries procedural risk, rendering such additional investigations inappropriate for pure research purposes.[8]. Therefore, more accessible, economic and time-effective means are required to usher novel imaging techniques from the bench to the bedside.

Realistic, in vitro data acquired from aortic flow phantoms represents one solution to such challenges [12]. Phantoms have become increasingly sophisticated in line with advances in imaging technology [12] alongside enhanced computation permitting replication of intricate anatomical structures using computer-assisted design (CAD). Moreover, the use of three-dimensional (3D) printing, a technology which has seen substantial growth in the healthcare industry, facilitates the manipulation of valve anatomy and control of the flow behaviour in turn [13, 14]. When coupled with accurate and robust ground truth pressure measurement, comparative data which is impossible to acquire in vivo can be obtained. This report details technical aspects of phantom fabrication, alongside reproducibility testing and the relationships observed amongst pressure metrics within the phantom.

### Aims of Haemodynamic Benchmark Creation

The following qualities would be essential for the phantom: (1) compatibility with both ultrasound (US) and magnetic resonance imaging (MRI), (2) plausible physiological mechanical (opening and closing) behaviour of the valve, (3) access to accurate ground-truth pressure measurement and (4) haemodynamic performance replicating in vivo pathology. Additionally, our work would provide a platform to start answering additional questions including which pressure metric within the phantom appears is most reproducible and how the various metrics relate to one another. The overarching aim of the phantom would be to provide data which could facilitate the refinement and validation of techniques for the haemodynamic quantification of AS. Such techniques, providing enhanced clinical risk stratification, patient selection and timing of intervention, would be of great use in an increasingly complex diagnostic landscape.

## Methods

### System Overview

**Fig. 1:**
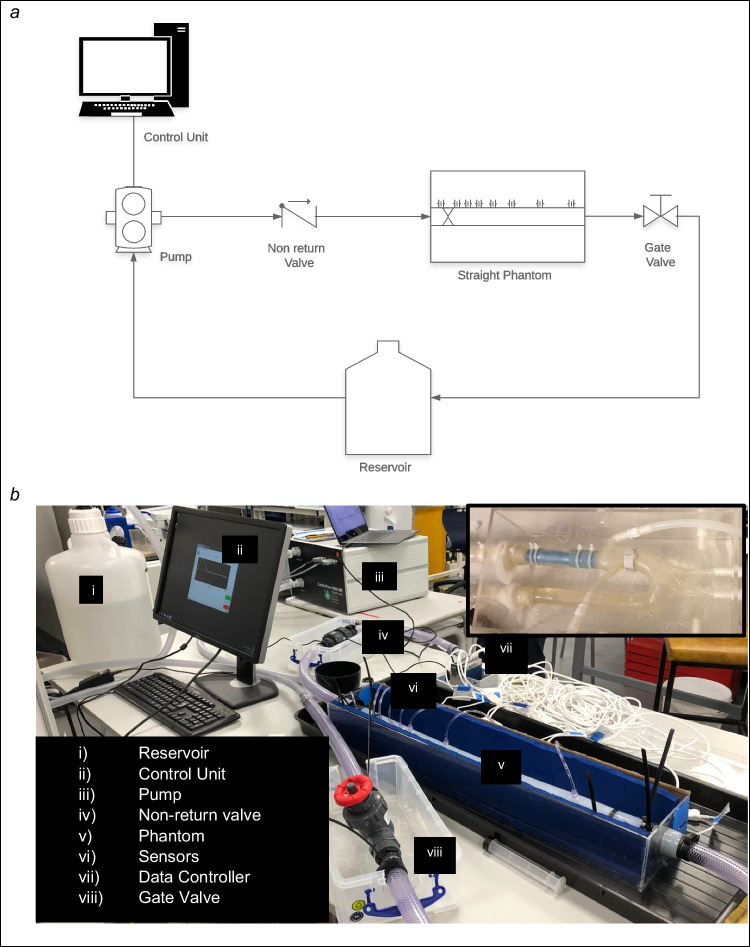
Schematic diagram of the phantom circuits and set-up. Panel a: common set of circuit elements. Panel b: picture of the straight aorta with sensors submerged in agar (circuit 1) with component parts listed in legend, inset: the anatomically correct aorta phantom (circuit 2)

The circuit consisted of a fabricated valve model, mounted into flexible tubing representative of the aorta, connected to a reservoir and a configurable flow pump (Fig. 1). Valve fabrication, along with a detailed discussion on material choice, is provided in the supplementary information.

### Ground-Truth Pressure Measurement

A custom system was designed and used to obtain an accurate direct pressure measurement. Eight flanged, female Luer lock to 1/16” barbed ports (Cole-Parmer, UK) were inserted into the phantom wall and secured using cyano-acrylate adhesive with silicone sealant applied to ensure robust attachment. Each pressure port is projected into the phantom thus resulting in a column of fluid in direct continuity with fluid in the “lumen” of the phantom. The pressure ports were located at fixed distances of −30, +15, +30, +50, +75, +100, +200 and +500 mm relative to the valve position.

Non-compliant 254 mm sections of 900 PSI-rated Luer-lock PVC tubing (30526-14, Masterflex, Oldham, UK) were attached to the ports allowing parallel connection of 8 PRESS-S-000 pressure sensors (PendoTech™, Princeton, NJ, USA). Each sensor was calibrated and validated against a solid-state pressure catheter (Mikro-Cath, Millar Inc, Houston, TX, USA). The output from the sensors was fed into a data controller (National Instruments, Austin, TX, USA), and the results were recorded using MatLab (MathWorks, Natick, MA, USA).

### Flow Circuit Fabrication

Two alternate circuits were used: circuit 1, a straight tube with attached pressure ports, and circuit 2, an anatomically accurate aorta without the pressure ports.

The idealised “straight aorta” in circuit 1 was created using a 750 mm section of 32 mm (internal diameter) silicone tubing (shore hardness 60) with a wall thickness of 3 mm. This section was suspended in a custom-made 4-mm acrylic box.

The phantom was connected to an MRI-conditional pulsatile flow pump (CardioFlow 5000 MR, Shelley Medical Imaging Technologies, London, Canada). The control unit allowed the alteration of various parameters of the pump output and provided the ability to recreate physiological flow. For the purposes of the investigation, a heart rate of 75 bpm was set for all data acquisition. The pump was calibrated to deliver given flow rates, and all parameters were kept constant except for the peak flow rate. The circuit started from the “flow-out” of the pump using a 2 m section of 16-mm pressure hose and travelled via a non-return valve (Spears, Sylmar, Ca) to the phantom. Beyond this, a shut-off valve was placed in series before fluid returned to the reservoir acting as an infinite compliance chamber, and then to the “flow-in” connection of the pump. The 20 L reservoir and phantom system were filled with a blood-mimicking fluid consisting of 60% distilled water and 40% glycerol with material qualities similar to blood (density of 1.119 g/cm^3^, viscosity of 4.83 × 10 to 3 Pa s and T1 value of 900 m s). A schematic representation of the circuit is depicted in Fig. 1.

An alternate circuit (circuit 2) (see Supplementary Material Figure 3 for circuit diagram) was created to better capture the physiological behaviour of the valve in an anatomically correct aorta. It has been described in detail previously [15]. The anatomically correct aorta, a commercially available compliant model (T-S-N 005, Elastrat, Geneva, Switzerland), was suspended within a custom-made 4-mm acrylic box. The phantom consisted of a complete aorta model including ascending, descending and coronary and brachiocephalic branches. The coronary vessels were clamped to ensure the flow of fluid was primarily down the aorta. The brachiocephalic vessel fluid convened into a shut-off valve that returned fluid directly to the reservoir. Fluid flowing out of the descending aorta ran into a custom-built compliance chamber. The valves were each mounted on a short section of HDPE pipe and inserted into the phantom such that the valve cusps lay at the level of the aortic annulus. This was perfused using the same pump and blood-mimicking fluid.

### Imaging Compatibility Assessment (Circuits 1 and 2)

A solution of 1% Agar was poured into the acrylic box and allowed to solidify, thus submerging the entire phantom and providing an US-conductive medium and static tissue comparison for phase contrast imaging. Both constant and pulsatile flows were used to acquire images. B-mode US images of the phantom were taken using a GE X95 US Machine using a 6S-phased array probe (GE Vingmed, Horten, Norway). Transverse images were obtained by insonation of the valve at an oblique, shallow angle (refer to Fig. 3 Dockerill et al. [16].). Longitudinal images were obtained placed with the probe parallel to the direction of fluid flow. Magnetic resonance imaging of the straight phantom was undertaken in a 3T scanner (Philips, The Netherlands) and for the anatomic phantom in a 1.5T Achieva (Philips, Best The Netherlands). Imaging was undertaken using the following parameters: field of view 200 × 200 × 114; reconstructed spatial resolution of 0.9 × 0.9 mm^3^; slice thickness 0.9 mm; TFE factor 2.

### Reproducibility Testing (Circuit 1)

Several conditions were created to assess each of the various factors which were expected to impact reproducibility. Condition 1 provided the baseline assessment of the valve. Condition 2 involved emptying the phantom of fluid, while maintaining a closed system and then refilling. The procedure to purge and refill the phantom would involve lifting and percussing sections of the tubing and the phantom itself. The positioning of the tubing after this step could introduce additional resistance in the system and alter the afterload conditions. Condition 3 involved purging the fluid from the phantom, dismantling the phantom and removing the valve, followed by replacing the same valve, reassembly and refilling. This could result in altered valve positioning and generate an error due to minor degrees of positioning or orientation of the valve relative to the pressure ports. Condition 4 was identical to condition 3 except that a different valve was placed into the phantom (valve *Y*—fabricated to be identical to valve *X*) which would indicate variability in valve manufacture technique. Each condition would involve the valve being subjected to four constant-flow and four pulsatile-flow regimens with peak flow rates of 100, 150, 200 and 250 ml/s. The pressure would be transduced directly using the pressure port sensors detailed above. These four conditions were undertaken on two distinct occasions, experiment A and experiment B, leading to a total of 8 conditions and 64 flow experiments (8 per condition).

### Analysis of Pressure Signals (Circuit 1)

Temporal transients of the 8 pressure sensors were acquired in each experiment. Each transient lasted for a few seconds (50,000 data points per channel, equating to 5–6 cycles in pulsatile flow). The raw pressure data was placed through a Butterworth filter to reduce noise.

The pulsatile pressure data was averaged over time to provide a single mean pressure tracing for each channel. The instant at which the peak pressure drop occurred was identified—channel 1 (before the valve) and channel 7 (after pressure recovery, and better than channel 8 due to spurious effects of valve reflections and remaining temporal components of the pressure drop) were selected. From this instant, the 8 samples of pressure along the phantom were used to reconstruct a pressure waveform by interpolation. The peak pressure drop was recorded as the maximum difference across time between the channel 1 and the interpolated value of pressure between the rest of the channels. The net pressure drop was measured as the difference between channels 1 and 7 at the time of the peak pressure drop. Details of the pressure analysis have been reported by our group[16]. The peak-to-peak pressure was identified as the absolute difference between the peak pressure measured in channel 1 and the peak pressure measured in channel 7.

The peak (maximal) and net pressure drops were extracted from the continuous pressure data following the same procedure.

## Results

### Phantom Compatibility and Versatility

**Fig. 2:**
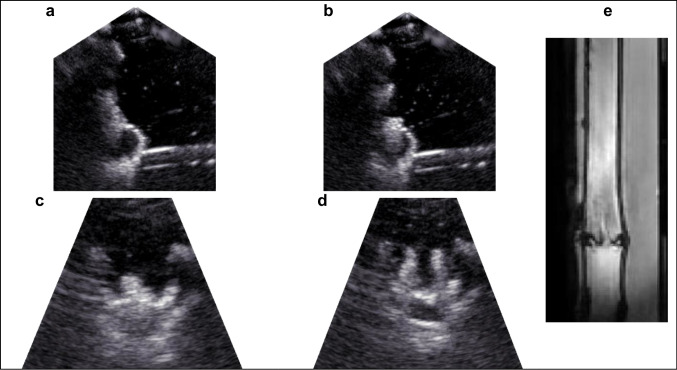
Images of a valve in closed (a and c) and open (b and d) conformation in US and in MRI (e)

**Fig. 3:**
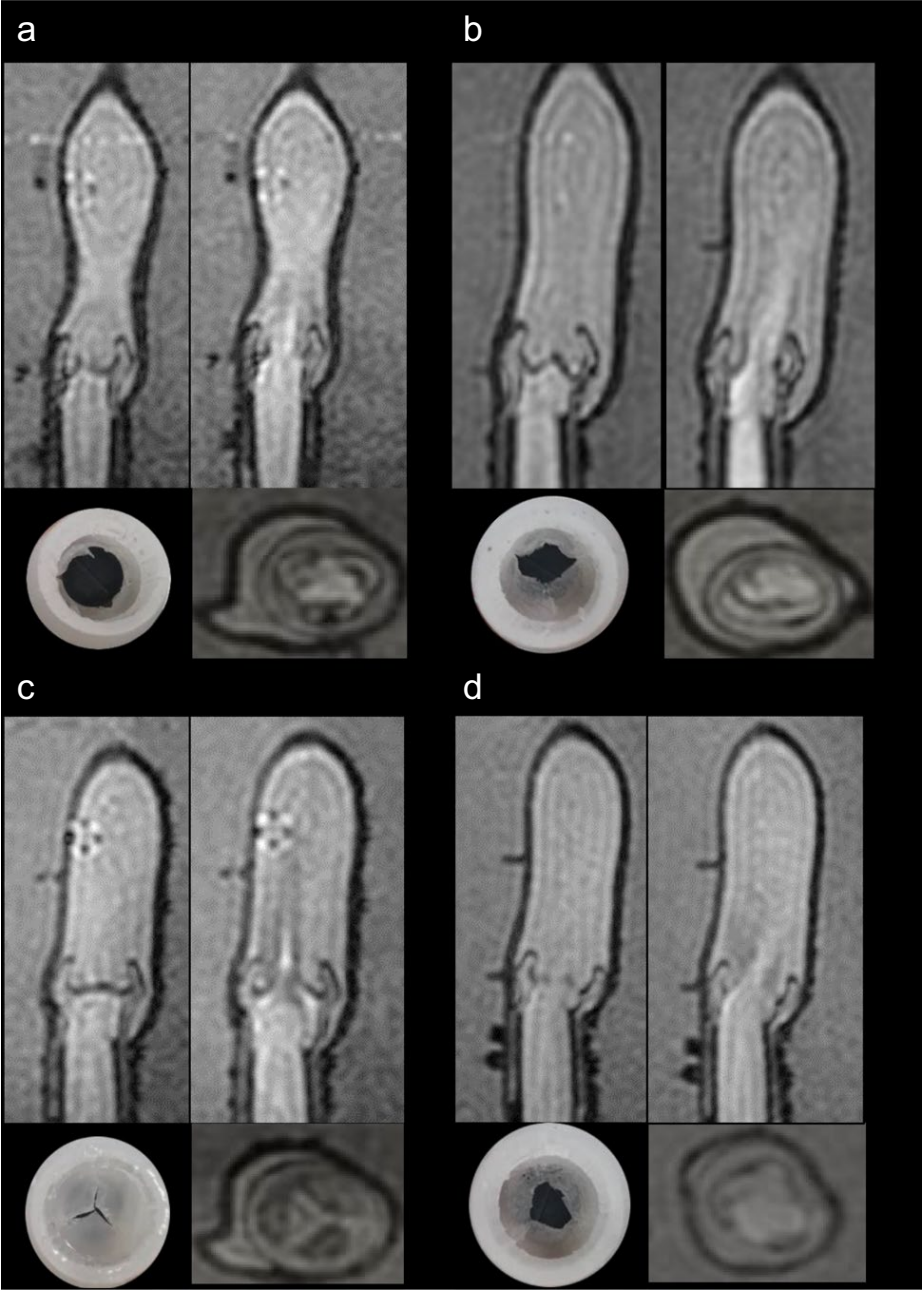
Images of the valves in circuit 2. In each sub-panel, the respective valve can be observed imaged by MRI in closed (left upper) and then open formation (right upper), en face (right lower) and finally the physical valve model (left lower). Four different valves are observed normal (a), bicuspid (b), calcific (c), rheumatic (d)

The phantom is both MRI and US-compatible. Figure 2 demonstrates the appearance of the valve in circuit 1 in both US and MRI settings. The US images reveal hyperechoic valve cups which open and close in a dynamic fashion. Blood appears hypoechoic, and the wall of the aorta is also reflective. Placed in circuit 2, simulated flow patterns captured by MRI imaging appear physiologically accurate and realistic (see Fig. 3): a healthy valve displays thin valve leaflets which open fully, and its main jet is located centrally; a bicuspid valve demonstrates unilateral restriction with incomplete opening and an eccentric jet; a calcific valve displays thickened cusps with severely restricted opening and a needle-like jet flowing through it; and a rheumatic valve shows a less distinct appearance of the valve cusps with incomplete closure and a reduced orifice size with a narrowed jet.

### Functional Testing: Pressure Waveforms and Pressure Drop Metrics

**Fig. 4:**
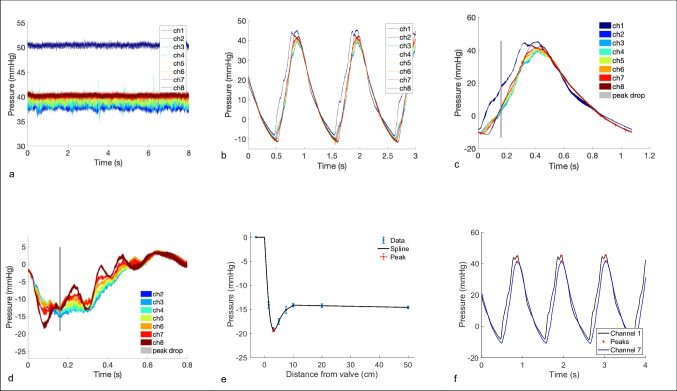
Graphical representations of pressure signals obtained from the sensors. Panel a: A typical time transient pressure recording under a single constant flow condition. Panel b: A typical time transient pressure recording under a single pulsatile flow condition. Panel c: Averaged pressure recordings for each channel under a single pulsatile condition, with the timepoint of the instantaneous peak pressure drop demarcated by a grey line. Panel d: Mean pressure drop between each channel and channel 1, with an overlay of the instant of peak drop (channel 1–7). Panel e: Reconstruction of the longitudinal transient of pressure through interpolation from data of the 8 sensor locations at the instant of peak drop (identified as shown in panel d), with an overlay of the estimated spatial location of the peak drop. Panel f: identification of peak-to-peak pressure drop under pulsatile flow as indicated by pressure transients in channels 1 and 7

A noisy pattern of pressure signals under constant flow conditions is captured (Fig. 4a), with the noisiest signals occurring in the closest proximity to the valve (channels 2 and 3).

Pulsatile flow curves demonstrate the expected separation of the pressure waveforms from channels 1–8 during the upstroke of flow to the peak flow rate, followed by a notched peak and then convergence (Fig. 4 b and c). The pressure waveforms across multiple beats display a very small variance, a reflection of a good beat-to-beat reproducibility (Fig. 4c). The extraction of the peak-to-peak pressure drop suffers from high-frequency oscillations in the peaks that may lead to unphysiological negative time shifts (Fig. 4d).

### Reproducibility of Different Pressure Drop Metrics

**Fig. 5:**
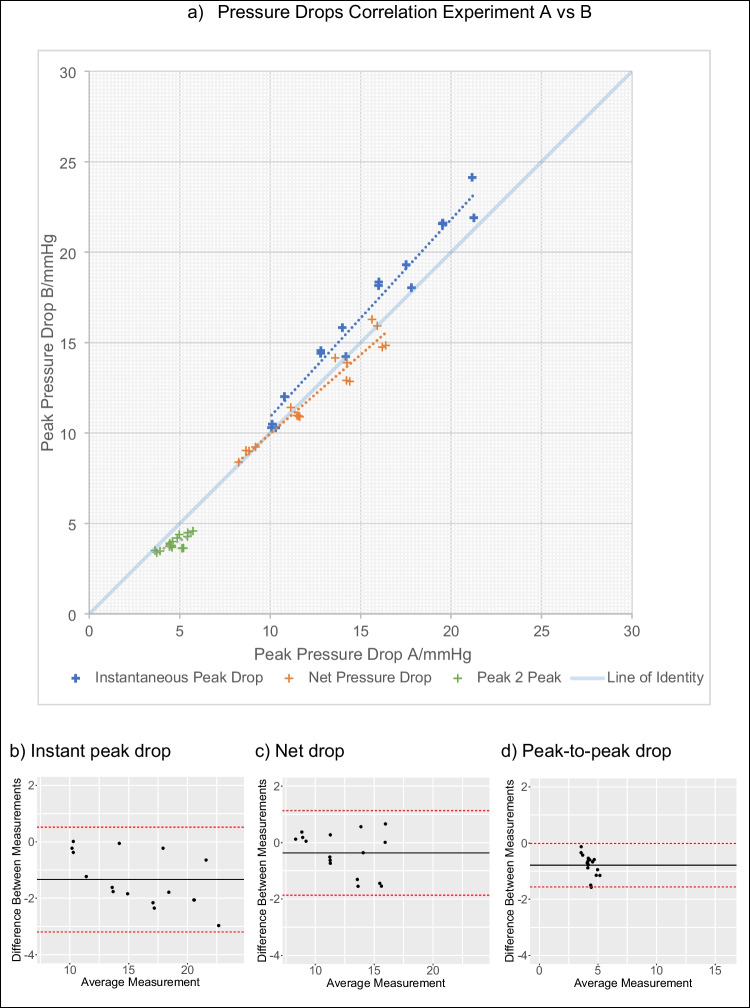
Reproducibility of pressure metrics in pulsatile conditions, comparing the measurements made in the two experimental sessions. Panel a: regression analysis. Panel b–d: the Bland-Altman analysis

**Fig. 6:**
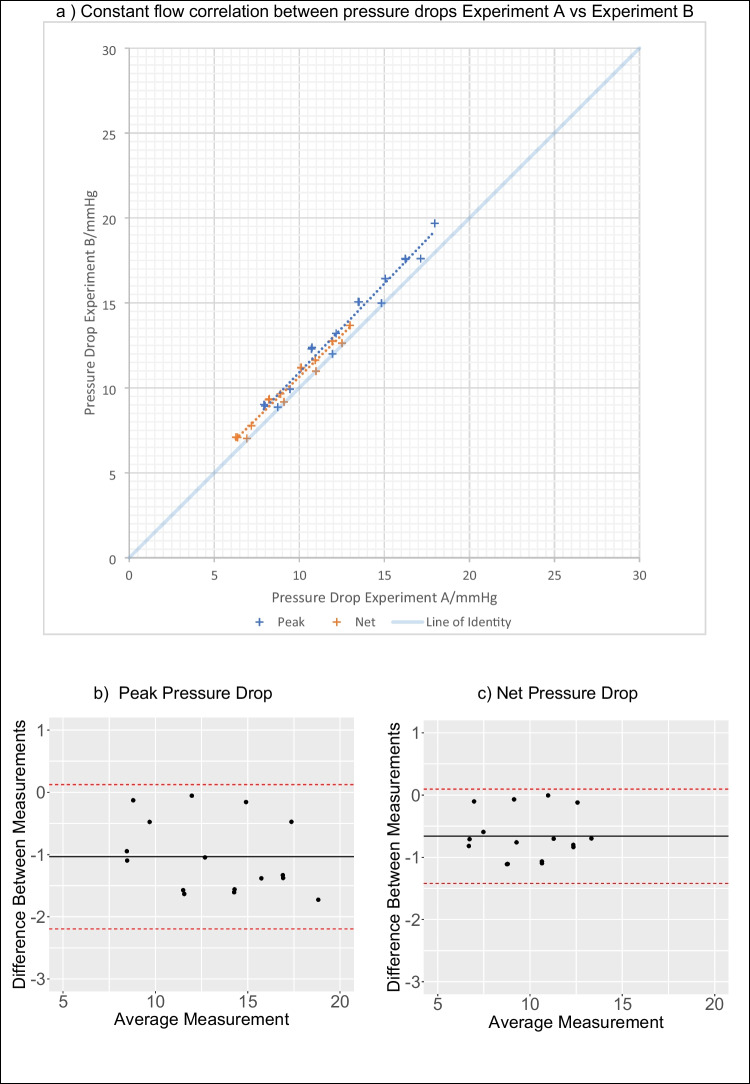
Reproducibility of pressure metrics in constant flow conditions, comparing the measurements made in the two experimental sessions. Panel a: regression analysis. Panels b and c: the Bland-Altman analysis

Whilst both the instantaneous peak and net pressure drop show a linear correlation between the different days of experiments and high levels of agreement *r*^2^ = 0.99 and 0.93 respectively, the peak-to-peak pressure drop showed a positive association with a weaker degree of correlation *r*^2^=0.61 (see Fig. 5). Constant flow conditions also reported similar excellent reproducibility of peak and net pressure drops (Fig. 6).

All pressure drop metrics did show a bias in between experimental days, displaying a small but appreciable increase in the obstruction of the valve in the second day. Under constant flow, a bias of +1.4 +/−0.59 mmHg (8.1%,) for the peak and +0.66 +/−0.39 mmHg (6.9%) for the net pressure drop was observed. Comparatively, with pulsatile flow, the bias was +1.34 +/−0.94 mmHg (8.7%), +0.36 +/−0.77 mmHg (3%) and +0.79 +/−0.40 mmHg (20%) for the peak, net and peak-to-peak respectively.

### Relation Between Pressure Metrics

**Fig. 7:**
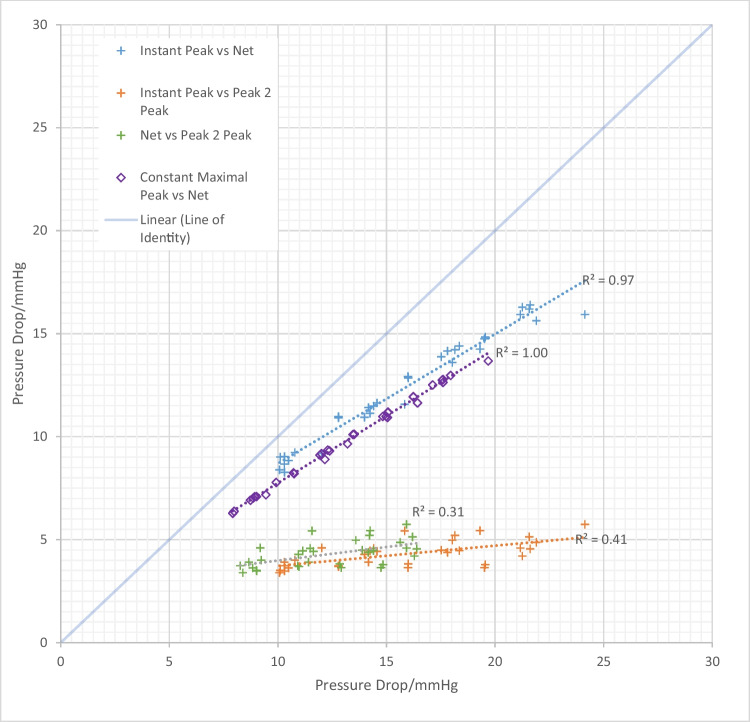
Scatter graph demonstrating the relationship between the various pressure metrics for pulsatile and constant flow

The instantaneous peak and net pressure drops did show an excellent agreement (*r*^2^=0.97 and *r*^2^=1.00 in pulsatile and continuous flow conditions respectively, see Fig. 7). The regression line reported the expected overestimation of the net pressure drop by the peak (regression coefficients of 0.63 and 0.65 in pulsatile and continuous flow conditions respectively). On the contrary, the relationship between the peak-to-peak with the peak and net pressure drops was quite poor (*r*^2^=0.41 and *r*^2^=0.31 respectively).

## Discussion

A physical phantom to emulate the condition of aortic valve stenosis with different grades of severity has been developed, and its physiological realism and imaging versatility have been demonstrated. The phantom reported that not all metrics of stenosis severity had the same reproducibility and that the peak-to-peak pressure drop may be affected by spurious factors not intrinsic to the valve.

### The Construction of an Aortic Valve Haemodynamic Phantom

In this report, we document a fabrication technique which yields functional and imaging-compatible aortic valves, created rapidly and at a low cost (see supplementary materials for an in-depth discussion of material choices and consideration). The phantom has already allowed the study of the US-based blood speckle imaging alternative for a more accurate estimation of the peak pressure drop [16] and the validation of an MRI-based method to estimate the pressure recovery distance (JCMR-D-22-00247 accepted for publication).

The silicone elastomer presented was a very cost-effective choice to generate a varying range of aortic valve flow conditions, and this has been reported previously [17]. The pressure drop observed across the healthy aortic valve model in circuit 1 is consistent with a non-diseased aortic valve. A peak velocity of > 2.5 m/s provides evidence to support a diagnosis of AS, which equates to a pressure drop of 25 mmHg by application of the simplified Bernoulli formula. Within our range of simulated flow rates, the instantaneous peak pressure drop remains below this figure with a maximum of 24.1 mmHg, indicating the success in recreating a non-pathological aortic valve. On the other hand, the other 3 disease aortic valve models mounted in circuit 2 did show the expected characteristics of narrower ejection jets.

An important quality was the compliance of the “aortic” wall. This deformability, as observed in vivo, contributes to the irreversible pressure drop across the valve and is a key factor in both health and disease [9, 18]. Previous phantom studies, which utilise rigid tubing, have lacked this additional variable when assessing valve haemodynamics [19–21].

A compliance chamber assists with simulating arterial compliance by replicating the reservoir function of the aorta and reduces the presence of wave reflections. The convergence of pressure waves during diastole amongst the channels distal to the valve (see panel c in Fig. 4) show the effectiveness in damping the wave reflections despite the absence of a dedicated compliance chamber.

### Sensing the Pressure Drops

Obtaining the ground-truth pressure measurement is challenging. Catheterized fluid-filled pressure sensors and pressure wires, used in vivo, display important limitations. Fluid-filled sensors display a dynamic range that can result in an overestimation of pressures [22], measurements that are subject to damping and resonance, and a reduced frequency response which inherently impacts temporal resolution [9, 23, 24]. Pressure wires have successfully been used for transaortic pressure drop measurement; however, the technology remains susceptible to difficulty with positioning and may also suffer drift causing uncertainty in the accuracy of measured values [25–27]. Our solution was direct pressure transduction from sensors in the phantom wall which were calibrated and validated against a gold-standard solid-state catheter. Eight simultaneous and accurately located sensors are a robust approach to the problem, limited by the need to interpolate along the length of the vessel to estimate the instantaneous peak pressure drop—our choice of a modified Akima piecewise cubic Hermite interpolation resulted in reasonable locations and magnitude of this metric (see Supplementary Figure 4 with the 32 reconstructed pressure waveform transients).

Constant flow yielded a noisier signal from sensors immediately close downstream to the valve. This noise is attributed to the physical vibrations/fluttering of the valve leaflets and flow turbulent oscillations in the proximity of the valve. Such effects were more prominent under constant flow, a regime that has the temporal conditions to fully develop the turbulent behaviour [28]. The fluttering of the valve tips was observed using the US in the constant flow conditions.

Pulsatile flow demonstrated less noisy signals and a generally smoother profile with a progressive upstroke pump to mimic the rapid emptying of the LV. Nevertheless, there was spurious high-frequency noise at key points of the pressure waveform (peak dp/dt or peak pressure)—these were interpreted to be an effect of the limited power of the pump because of their very localised nature, its consistency across channels and experimental conditions, and the fact they were modulated by the pump settings (attempts to deliver larger and steeper pressure waveforms will result in larger localised noised events). The pressure flow profile adopted was chosen to minimise these effects, but they were still present and were one of the secondary factors contributing to the negative time shifts in the computation of peak-to-peak pressure differences.

### Testing Reproducibility: Expected and Unexpected Findings

The high correlation of the peak and net pressure drops (*r*^2^>0.93) are likely representative of the high level of control in the workbench set-up and testing protocols which minimise confounding effects. The instantaneous peak pressure drop is observed to have the greatest correlation in the test-retest amongst the metrics. This is despite the use of interpolation to estimate the spatial location and magnitude of the peak pressure drop which is usually observed within 50 mm of the valve (between channels 2 and 3). Reassuringly, this high degree of reproducibility was maintained amongst two different identical valves (correlation between control with valve *X* vs valve *Y r*^2^ =. 0.995, CI 0.976–0.999, *P*<0.000001) highlighting the consistency of valve fabrication technique (see supplementary materials). The physical distance between the sensors gives greater scope for variability amongst additional factors such as jet angulation, aortic wall deformation, oscillation and reflected waves which summate to increased variability in the recovered pressure recorded. All these factors were controlled for and only contributed to a marginally lower correlation.

On the contrary, the peak-to-peak pressure drop displayed a low reproducibility (*r*^2^=0.61), strongly suggesting that this signal is sensitive to other factors that are not intrinsic to the obstruction generated by the valve. The inspection of the morphology of the pressure waves at the different channels reveals the presence of pressure augmentation caused by the reflection of the pressure wave at the end of the phantom aortic tube, as it happens in the human adult aorta (at points of impedance mismatch) [29]. The addition of the forward and backward travelling waves is interpreted to cause the apparent negative shift of the peaks of the pressure waves (see panel c in Fig. 4). The peak of the pressure signal, and thus the peak-to-peak pressure drop, are therefore also ruled by the coupling conditions of the vascular system (i.e. wave reflections), and these are factors that we did not manage to properly control for in our phantom.

All pressure drop metrics did show a positive bias in the second day of experiments compared to the first one. It is interpreted that this may be caused by degradation due to interaction with the phantom fluid causing reduced compliance or increased friction at the leaflet surface, although there is no evidence in the literature which supports this hypothesis. Moreover, the phantom was stored in a purged state between experimental runs, reducing the likelihood of significant material interaction. An alternative explanation could be a systematic zeroing error at the time of data acquisition to result in a consistent bias throughout the experiment.

The pressure drop for a given flow rate was greater for pulsatile flow when compared with constant flow. This finding might be regarded as unexpected, since both conditions (pulsatile and constant) were designed to deliver the same peak flow rate. It should be noted that the average flow rate is much larger in the constant flow condition. However, the average flow does not influence the peak events being characterised across the 4 different flow regimes which should ultimately lead to the same spatial acceleration that rules the peak pressure drop (Donati et al. 2017). The difference observed is attributed to the transient nature of the pulsatile flow: it does not have enough time to build the steady (and more efficient, valve fully open) flow condition of the constant flow. Another potential explanation would be the deformation of the valve to yield a larger orifice area under constant flow. However, this phenomenon was not observed during real-time US imaging.

### The Metrics that Characterise the Haemodynamic Burden and Clinical Perspectives

When considering the pressure drops across the valves, they can be broadly dichotomised into either peak (or maximal) or net pressure drops. Peak pressure drops can be calculated at a temporal instant or at distinct time points, from DE (instant peak) and catheterisation (peak-to-peak) respectively. The net pressure drop refers to the recovered pressure measured at a sufficient distance distal to the stenosis to ensure that the energy temporarily converted into kinetic energy is recovered as the blood flow achieves complete deceleration. The net pressure drop best characterises the additional haemodynamic burden faced by the ventricle in respect of the obstruction to flow[30]. On the other hand, the peak instantaneous pressure drop (which is derived from the peak velocity in clinical practice) is a specific metric of the valve obstruction, effectively a localised measure of acceleration at the valve level. Its reliability in our phantom results, together with the excellent agreement with the net pressure drop, continued use for clinical practice, especially for valve surveillance given the high level of reproducibility.

Within our phantom, the peak-to-peak pressure drop was not well correlated with the instant peak or net pressure drops. This is explained by the fundamental difference of these pressure difference metrics, the former being an asynchronous peak-to-peak (and thus also affected by changes in absolute pressure at two different time points such as those dictated by wave reflections) and the latter being instantaneous (and thus entirely ruled by the physics of the Navier-Stokes equations) [29]. In clinical practice, the peak-to-peak measurement via catheter pullback of pressure drops is discouraged for grading of AS, and our experimental findings support this [1].

The observation that instantaneous and net pressure drops are less well correlated under pulsatile flow is an important one. It signals that the unsteady nature of pulsatile flow introduces additional variability to the system and provides some explanation for the difficulty faced in estimating the net pressure drop from peak pressure drops.

Whilst the instantaneous peak and mean pressure drops derived from DE are important for clinical decision-making, the historic gold standard has been the invasive mean pressure drop [31] as it accounts for account pressure recovery. The net pressure drop is influenced not only by the stenotic valve, but also by the aortic diameter, geometry, jet angulation and the effects of systemic hypertension. From a clinical standpoint, such a measure has excellent correlation with outcomes. Our measure of the net pressure drop—the recovered pressure at the time of peak velocity recording—has no clinical parallel, although simultaneous left ventricular and aortic invasive pressure recordings would be able to provide the equivalent data.

### Limitations and Further Work

Our pressure results were studied only with a healthy valve design, where an appreciable but clinically small pressure drop was observed. Further evidence with higher stenotic conditions is warranted before extrapolation to pathological valves can take place. Additionally, the symmetry of valve opening and the subsequent development of eccentric flows was not controlled for or evaluated. Evidence has demonstrated that eccentricity contributes to inefficient flows that impact haemodynamics, and this should be subject to further investigation. As with any model, the fidelity of our phantom is limited (e.g. a homogeneous silicon material was used to mimic a complex composite tissue as discussed in detail in section “Material Considerations and Choices” in the supplementary materials), and as such, the generalisation of our findings in real patients cannot be inferred. Whilst in vivo pressure drops are governed by a complex interplay between factors including the ventricular function, the valve obstruction and systemic arterial compliance, our circuit only focussed on the role of the valve. Therefore, system-level conclusions cannot be made without reservation. Specifically, the impact of wave reflections in the peak-to-peak pressure drop warrants further investigation.

## Conclusion

A flow phantom compatible with MRI and US, demonstrating plausible valve opening and closing both in healthy and pathological conditions and with access to ground-truth pressure measurement, has been built and successfully tested for control and reproducibility. Across current metrics of the aortic stenotic burden, the peak-to-peak pressure drop appears confounded by valve-unrelated factors such as wave reflection and should thus be reconsidered in clinical practice.

## Supplementary Information

Below is the link to the electronic supplementary material.

Supplementary file1 (DOCX 1632 KB)

## Data Availability

The datasets generated during and analysed during the current study are available from the corresponding author on reasonable request.

## Abbreviations

AS: Aortic stenosis
CAD: Computer-assisted design
DE: Doppler echocardiography
EOA: effective orifice area
LV: Left ventricle
MPD: Mean transaortic pressure drop
MRI: Magnetic resonance imaging
US: Ultrasound
Vmax: Peak aortic velocity
