# Supplementary material for: Aortic Stenosis: Haemodynamic Benchmark and Metric Reliability Study
Source: J Cardiovasc Transl Res. 2023 Feb 6;16(4):862–73. doi: 10.1007/s12265-022-10350-w (PMC10480252; doi:10.1007/s12265-022-10350-w)
Supplement: Supplementary file 1 — Supplementary file1 (DOCX 1632 KB) [file 12265_2022_10350_MOESM1_ESM.docx]

Supplementary Information

## Valve Fabrication

Manual segmentation of a cardiovascular computed tomography scan of a healthy aorta, aortic valve and left ventricular outflow tract was undertaken using open-source, purpose-built software ITK-SNAP (www.itksnap.org). Three dimensional meshes were extracted using this program in stereolithography (STL) format and then transferred to SolidWorks (Dassault Systems, Waltham, MA). The CAD valve model was straightened and simplified to optimise the structure for 3D printing (see *Supplementary Figure 1*).

*Supplementary Figure 1 a) Internal and external mould pieces, b) internal mould printed in PVA, c) assembled mould after silicone injection, d) blueprint valve prior to refinement*

| a | c |
| --- | --- |
| **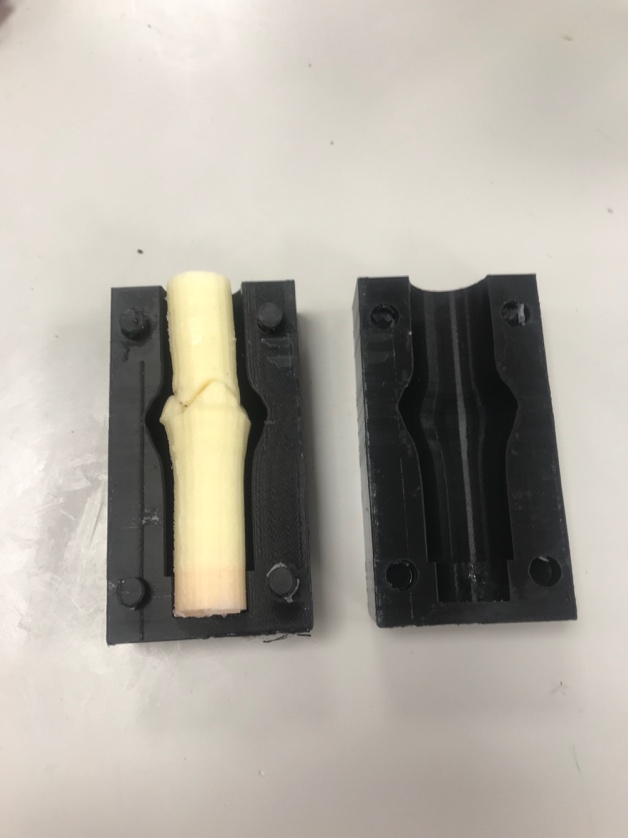** | **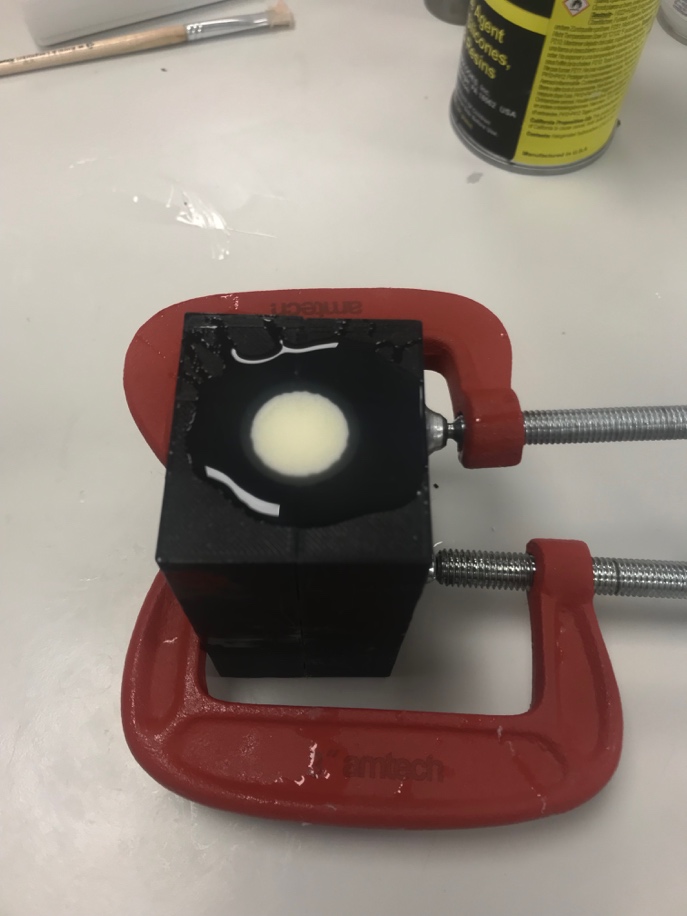** |
| b | d |
| **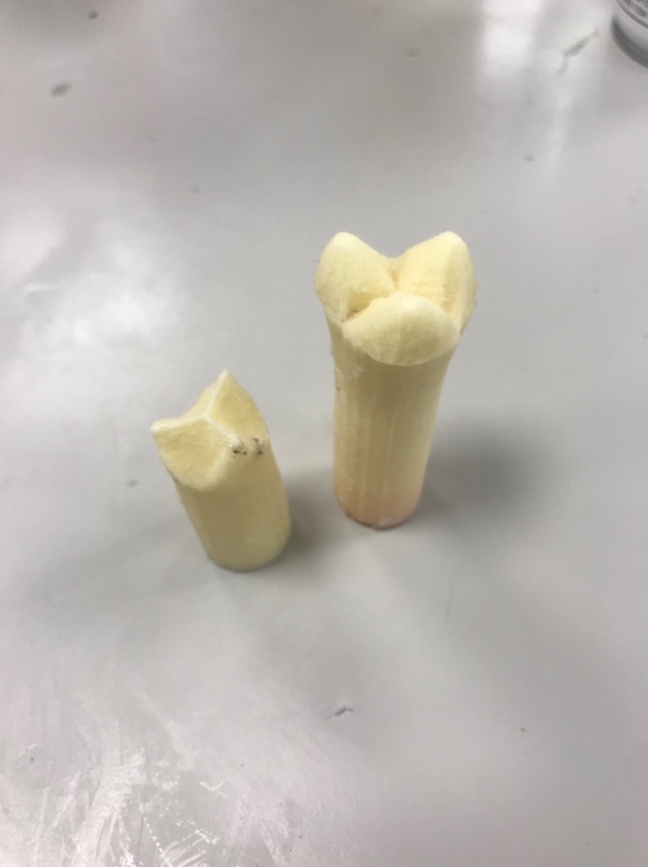** | **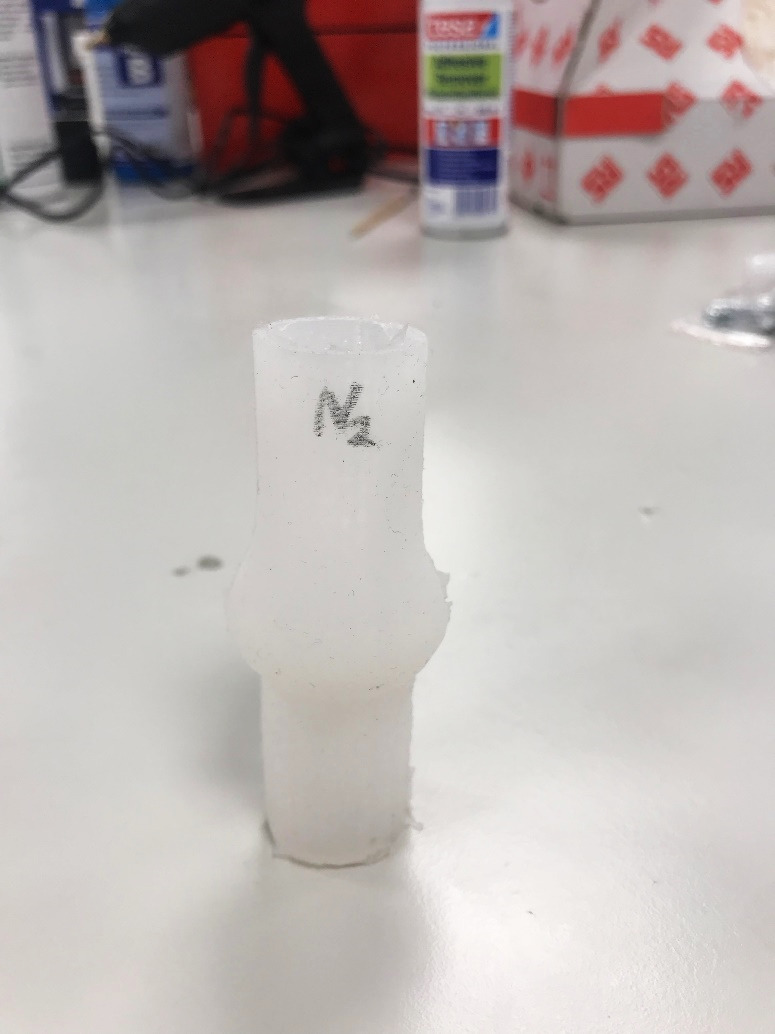** |


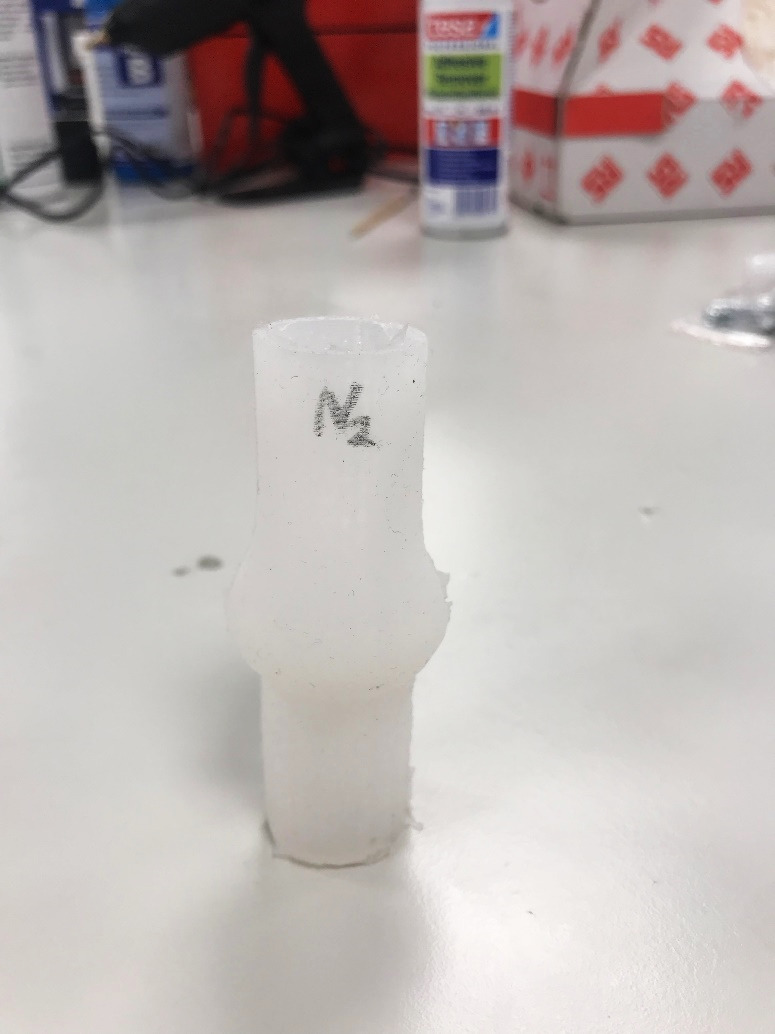
A multi-part mould consisting of two external pieces and two internal pieces was then designed. Specialist slicing software (Cura 3D, Ultimaker, Utrecht, The Netherlands) was used to organise the STL file into gcode format suitable for fused filament printing. Settings were adjusted to give the optimum balance between print quality and time. The external pieces were printed in rigid polylactic acid (PLA) and the internal pieces were created in a more flexible polyvinyl alcohol (PVA) material. All support material was removed from the PLA to ensure a smooth external structure. The PVA internal mould was filed to remove any rough edges on areas adjacent to the valve leaflets which could disrupt their formation. Silicon release agent (Ease Release 200, Smooth On, Macungie, Pennsylvania, USA) was applied to all the internal surfaces of the mould prior to assembly. Manual clamps were secured on the outside of the mould to ensure silicone did not infiltrate the seam lines between the adjacent parts. Ecoflex Silicone 0030 (Smooth-On, Macungie, Pennsylvania, US) is a commercially available two part, room temperature-cure silicone and this was combined according to the instructions in a 1:1 part A:B ratio by mass. The resulting mixture was stirred together for 5 minutes, before being subjected to a vacuum chamber to degas the liquid for a further 10 minutes. The resulting liquid silicone mixture was poured into a 50ml plastic syringe and injected into the mould. The mixture was cured over 24 hours and the valve model was then removed from the mould. The PVA internal mould, although originally intended to be dissolved, was conserved and re-used to maximise reproducibility and reduce the overall fabrication time to under 26 hours.

Four valve types were generated using the steps described above and refined to simulate a range of physiological scenarios. A “normal valve” was created by complete dissection of the valve cusps along the coaptation lines (note that valve cusps will be fused after the manufacturing process). Three typical AS pathologies were then created: a bicuspid valve with a single coaptation line left fused; a rheumatic valve replicated the circumferentially fused cusps by partial dissection of the one third of the coaptation lines; and a calcific, degenerative valve displayed uniformly thickened valve cusps and increased resistance to opening by adding an additional 1cm^3^ of Ecoflex™ 0050 to the valve cusps.

A system was designed to enable the mount and exchange of valves. For each valve the portion of silicone distal to the valve beyond the equivalent of the sinotubular junction was trimmed off. Each of the resulting valve models were mounted using a cyano-acrylate adhesive within a short 3D-printed PLA bracket to permit easy exchange of valves.

Material Considerations and Choices

Human aortic valves are composed of an underlying fibroelastic structure encased in a non-thrombogenic layer of non-vascular endothelial cells[35, 36]. Gross aortic valvular anatomy and the intricate microarchitecture are not easily recreated in synthetic materials[37]. The fibroelastic scaffold is flexible and compliant enough to deform in the setting of flow through it[35], but is also endowed with elastic properties which allow complete return to the pre-deformed state. The smooth valvular surface is essential to maintain efficient fluid flow for healthy valve function which remains constant over thousands of cycles. Our aim was to create a model of the aortic valve, replicating these properties while using equipment already available within the engineering facility, at a reasonable cost.

Analysis of potential materials showed few possessed tissue-like qualities with even fewer suitable for direct 3D. Despite successful fabrication of a functional patient-specific valve model using TangoPlus (Stratasys, MN, USA). Alongside non-desirable acoustic properties of TangoPlus preventing US imaging, alternative material were sought. Room temperature vulcanising (RTV)-silicones are well imaged using US and have an elasticity matching that of blood vessels[38]. RTV-silicones such as the Ecoflex™ range of silicones (Smooth On, Easton, PA, USA) are highly compliant with a Young’s modulus of 0.0178-0.0965 MPa, lower than reported uniaxial Young’s modulus testing of native valve tissue. Whilst canine aortic valves have been reported to have a radial Young’s Modulus of 2.4-52 MPa throughout the cardiac cycle, the stress-strain curves were non-linear highlighting the complexity of valve function. Direct tissue testing for excised porcine aortic valves revealed an average Young’s Modulus of 1.33 MPa and in human data the figure ranges between 1.57-7.50 MPa for uniaxial radial testing. Subsequent data modelling of the stress-strain curves in porcine valves confirms the non-linearity of the relationship[39]. The non-linearity is a result of the composite histological structure of the valves, and anatomy which both contribute to the deformability and elastic properties of the valve. This is something that is not easily mimicked when using a single material and this underlines the success in creating physiologically functioning valves with plausible haemodynamics.

Comparing valve fabrication

The following scatter plot demonstrates the comparison of the control condition on each day against the condition where valve X was exchanged with valve Y (*Supplementary Figure 2*):

*Supplementary Figure 2: Scatter graph showing the correlation between the control condition pressure drop of valve X against valve Y.*

The instantaneous peak pressure drop is compared from the control condition (condition 1) were compared to the replacement of valve X with valve Y (condition 4).

*Supplementary Figure 3*

*Schematic diagram of Circuit 2 with the anatomical aortic phantom and additional compliance chamber.*


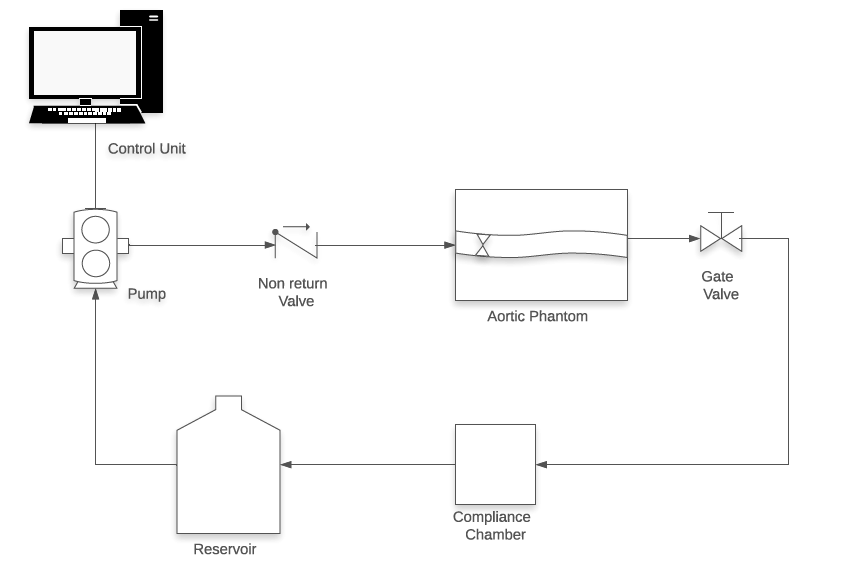


*Supplementary Figure 4*

*Instantaneous Peak Pressure Drop Transients for pulsatile flow*

| Flow rate | Condition 1 | |
| --- | --- | --- |
|  | Experiment A | Experiment B |
| 100 |  |  |
| 150 |  |  |
| 200 |  |  |
| 250 |  |  |

| Flow rate | Condition 2 | |
| --- | --- | --- |
|  | Experiment A | Experiment B |
| 100 |  |  |
| 150 |  |  |
| 200 |  |  |
| 250 |  |  |

| Flow rate | Condition 3 | |
| --- | --- | --- |
|  | Experiment A | Experiment B |
| 100 |  |  |
| 150 |  |  |
| 200 |  |  |
| 250 |  |  |

| Flow rate | Condition 4 | |
| --- | --- | --- |
|  | Experiment A | Experiment B |
| 100 |  |  |
| 150 |  |  |
| 200 |  |  |
| 250 |  |  |
